# Self-Efficacy in Breast Cancer Patients: A Pre–Post Study of a Brief Digital Psychosocial Intervention

**DOI:** 10.3390/diseases13070199

**Published:** 2025-06-28

**Authors:** Dimitrios Charos, Maria Andriopoulou, Giannoula Kyrkou, Anna Deltsidou, Glykeria Vaina, Victoria Vivilaki

**Affiliations:** 1 General Anti-Cancer Oncology Hospital AgiosSavvas, 11522 Athens, Greece; 2 “Konstantopouleio” General Hospital of Nea Ionia, 14233 Athens, Greece; 3 Midwifery Department, University of West Attica, 12243 Athens, Greece; 4 Mobile Mental Health Unit of Kefallonia, Zakynthos and Ithaki, 28100 Kefallonia, Greece

**Keywords:** breast cancer, family support, self-efficacy, family communication, brief digital intervention

## Abstract

Background: Breast cancer significantly impacts the social relationships and self-efficacy of affected patients. Purpose: To investigate the role of self-efficacy and the ability to maintain social relationships in breast cancer patients during the postoperative period. Method: This study is a brief intervention study in the same population group (within-subjects intervention study), in two measurements (pre-test and post-test), conducted in 58 breast cancer patients hospitalized in oncology hospitals in Athens (February 2021–November 2021). The following validated scales were used: the Social Relationship Coping Efficacy Scale (SRCE), the Family Support Scale (FS-12), and the Family Problem Solving Communication Scale (FPSC). Results: The mean age of the participants was 52 years. No statistically significant differences were observed in the scales after the intervention. The degree of change in the scales had minimal differences across all types of treatment. However, there was a statistically significant correlation between the change in the SRCE and the FPSC (*p* = 0.043), which suggests that the improvement in the ability to maintain social relationships is related to the strengthening of family communication. Conclusions: The intervention had a positive effect on maintaining social relationships and improving communication for problemsolving ability, although the overall changes in the scales were not statistically significant.

## 1. Background

Breast cancer has increased significantly in recent years and prognostic data indicate that it will increase even more in the future. It is the most common form of cancer in women worldwide with increasing survival rates due to advances in diagnosis and treatment [1].

Despite this progress, the disease continues to have a significant impact on both the sociability of patients and the effectiveness of maintaining their social relationships. Mastectomy, in particular, negatively affects the psychological well-being, self-image, and social relationships of patients. Social isolation caused by breast cancer can negatively affect the quality of life of both the patients themselves and their family caregivers [2].

Specifically, women who undergo mastectomy are particularly vulnerable during the preoperative and postoperative periods. It is a critical period for both patients and their families, due to the high vulnerability of the patients. Patients, mainly due to the mastectomy and the start of their treatment, experience high rates of anxiety, stress, difficulty adjusting, fear of treatment, fear of recurrence, etc. [3,4]. In this context, psychosocial interventions are particularly comforting [5,6] and even brief psychosocial interventions can have positive effects on patients’ psychosocial adjustment [7,8,9].

Social and family support are critical factors in adapting to the disease, its management and treatment [10], and improving the mental health and quality of life of patients [11].

The level of social and family support of patients with breast cancer plays a decisive role in preventing emotional distress and the onset of depression [5,12,13,14], contributes significantly to the relief of depressive symptoms and anxiety [15], and improves the quality of life of patients [16]. Social support for patients plays an important role in reducing social isolation and at the same time improving anxiety and depression. On the other hand, family influences patient support, and for this reason, close family relationships positively affect patients and improve their quality of life [13]. Tangible support improves the physical quality of life of patients, while positive social interaction is related to all areas of quality of life [17].

A fundamental psychological concept closely related to adaptation to chronic illness is self-efficacy. It is the individual’s perception that he or she can successfully cope with demanding or stressful situations. According to Bandura’s (1982) theory [18], self-efficacy is shaped through four main sources: personal experiences of success, observation of models (learning imitation), verbal encouragement, and emotional arousal. These factors influence the way an individual thinks, acts, mobilizes, and responds emotionally to stressful situations.

In the field of oncology, self-efficacy is associated with positive outcomes for patients, such as greater adherence to treatment, increased self-care behaviors, better psychological adjustment, and improved quality of life [19,20,21]. Research shows that high levels of self-efficacy are associated with reduced anxiety and depression, greater mental resilience, and higher levels of emotional stability [21,22]. Conversely, low levels of self-efficacy are associated with increased levels of emotional distress and difficulties coping with the disease [22].

In an attempt to measure this parameter in the oncology population, the SRCE (Social Relationship Coping Efficacy) scale was developed by Merluzzi et al. (2019) [23] and validated in Greek by Charos et al. (2021) [24]. This scale measures the patient’s perceived ability to maintain and manage social relationships during the course of the illness.

Despite the increasing emphasis on the importance of self-efficacy and social support in the context of oncology care, the literature remains limited in evaluating brief, digitally delivered psychosocial interventions in women with breast cancer during the perioperative period. This is an extremely critical and vulnerable phase of the disease, in which patients’ needs for psychological support are increased but often not adequately met.

This study attempts to fill this research gap by examining the effects of a brief, structured, digital psychosocial intervention on self-efficacy and family communication in women with breast cancer undergoing mastectomy, designed to be easily implemented by health professionals in the early postoperative period, even in remote areas.

At the same time, it explores the association between strengthening social relationships and problem-solving skills in the family context. The intervention incorporates elements of empathy, psychoeducation, and mobilization for support from the family and social environment, and is systematically implemented three months after surgery.

This study is original in that it implements a ten-minute psychosocial intervention remotely, with clear content and structure, to women who are in a particularly vulnerable phase of their treatment course. The intervention is evaluated using weighted instruments, in a pre- and post-intervention design, within a homogeneous sample, which provides increased internal validity.

Finally, this study aims to investigate the effects of a brief digital psychosocial intervention on self-efficacy and family communication in patients with breast cancer.

## 2. Materials and Methods

### 2.1. Study Design

This was a within-subjects pre–post study, conducted among breast cancer patients during the preoperative and postoperative period. The design involved two measurement points—baseline and three months post-intervention—with no control group. The participants were informed about the purpose and procedure of the study, and it was made clear that the participants retained the right not to participate in the study or to discontinue participation without any impact on the care provided for their treatment.

In the first part of the study (during the preoperative period), the SRCE, FS-12, and FPSC questionnaires were administered in person at the time of patient admission.

In the second part of the study, three months after surgery (postoperative period), the researcher had telephone contact with the patients participating in the study where he carried out an intervention by asking questions regarding awareness, psychoeducation, and information. The participants then answered the questionnaires in electronic form. A specific framework and time frame were set for both the intervention and the completion of the questionnaire.

More specifically, the intervention was carried out simultaneously with the second measurement via telephone communication. Immediately after its completion, the questionnaires were sent electronically to the participants for completion. The participants had a time limit to complete the material (within two days). In this way, it was ensured that the second measurement directly reflects the result of the intervention.

This study applied a within-subject intervention design, without the use of a control group. The same participants were assessed at two time points (before and after the intervention), and there was no allocation to different research groups. Of the 70 patients initially included, 58 completed both phases of the study. The loss of 12 participants was due to either inability to contact during the second phase, or late or incomplete response to the questionnaires, according to the pre-specified exclusion criteria. The response rate including the second measurement was 0.82%.

The recruitment period lasted from February 2021 to November 2021. An extension of this period was not possible, as health restrictions imposed due to the COVID-19 pandemic (particularly in oncology hospitals) limited access to inpatient clinics. In addition, completing recruitment within this timeframe was deemed necessary to ensure the required follow-up of participants three months after surgery and at the same time allowed for the smooth conduct of the study phases.

As the study was implemented under particularly restrictive conditions during the COVID-19 pandemic, no statistical sample size calculation was performed. The health restrictions that applied in oncology hospitals, combined with the psychological burden of patients, significantly limited the possibility of accessing, recruiting, and following up with participants.

However, statistical significance and effect sizes are presented in detail in the results to support the statistical and practical importance of the findings. Furthermore, the study aims not to generalize to the general population, but to initially assess the impact of the intervention in a real hospital environment and in patients with common characteristics and clinical picture.

Therefore, the study was designed with realistic research objectives based on a number of participants that was deemed feasible, functional, and comparable to similar research efforts under crisis conditions. The number of 58 completed participants provides sufficient data for an initial assessment of feasibility, acceptability, and first indications of effectiveness, while the findings can be used to design subsequent larger-scale studies.

The study has a within-group design (pre–post, without a control group) and its main objective was to investigate the effect of a specific intervention on psychological variables, such as self-efficacy and family communication.

### 2.2. Participants

The sample consisted of female patients with breast cancer, selected based on specific inclusion and exclusion criteria, in accordance with the guidelines for interventions in oncological populations. The sample was recruited from public oncology hospitals in Athens, during the period February 2021–November 2021, after permission was granted.

The inclusion criteria for participants in this study were as follows:

- Women aged >18 years, diagnosed with breast cancer.
- Hospitalized patients, scheduled for mastectomy.
- Patients should have access to digital means of communication.
- Be fluent in Greek.
- Be able to read and write.

The exclusion criteria were as follows:

- Women with a history of serious psychiatric disorders.
- Not completing the study or delaying answering the scales too much.
- Having metastatic cancer.

The questionnaires were given to a random sample of 70 hospitalized women with breast cancer who were about to undergo surgery.

The selection of hospitalized women with planned mastectomy as the target population was based on the international literature, according to which the preoperative and early postoperative periods are particularly sensitive time points in terms of psychosocial burden and the need to strengthen adaptation mechanisms. This specific group allows for the immediate provision of support in a phase of increased anxiety and vulnerability and at the same time offers research homogeneity, as the patients are at a similar stage of the disease and their treatment plan. Finally, this vulnerable period of breast cancer patients has not been extensively researched by the scientific community.

Although 70 patients were initially recruited, only 58 completed both the pre- and post-intervention assessments. Thus, the final response rate was 82%.

### 2.3. Measurements

The following validated scales were used in this study:

Social Relationship Coping Efficacy Scale (SRCE) [23]. The SRCE consists of 10 items and is based on the theory of self-efficacy. It assesses the individual’s ability to demonstrate behaviors that maintain or enhance social support and social relationships. It is a Likert-type scale, ranging from 1 (not at all confident) to 9 (completely confident). The Cronbach α of the Greek version of the SCRE was α = 0.87 [24].

FamilySupportScale (FS-12).Created in Finland by Julkunen et al., 1989 [25] and assesses the subjective feeling of patients who are supported by their family and diagnosed with a chronic disease. The questionnaire consists of 12 items, is a Likert-type scale, ranging from 1 to 5, and the Cronbach’s alpha of the Greek version FS12GR was α = 0.77 [26].

Family Problem Solving Communication (FPSC). Developed by McCubbin et al., 2001 [27] and assesses the ability to communicate in the family with the ultimate goal of solving problems and conflicts in the family. The FPSC has a Cronbach α = 0.89 [27,28].

### 2.4. Data Collection Procedures

Data collection was carried out in two time phases in order to cover the period before and after the intervention. The first phase concerned the time before the surgery, while the second was carried out three months after the surgery. The aim of this structure was to capture changes in the studied psychosocial indicators.

First phase—preoperative period:

During the admission of the patients to the oncology hospital, information was provided regarding the purpose of the research and written consent was requested from the participants. Subsequently, the three psychometric instruments (SRCE, FS-12, FPSC) were completed in person before the surgery.

Second phase—three months after the surgery:

The same participants were called by telephone by the researcher. During the telephone contact, a brief 10 min psychosocial intervention was implemented, based on a predefined protocol (as described in Section 2.5).

Immediately after the intervention, participants received an email link to re-complete the same assessment tools, in electronic format (Google form). A two-day time limit was set for submitting responses to ensure that the measurement reflected the immediate effect of the intervention.

For standardization reasons, all interventions were carried out by the same researcher. Of the 70 women who participated in the initial phase, 58 completed both phases of the study. The exclusion of 12 cases was due to non-response, inability to communicate, or delay in submitting the tools beyond the specified time frame.

The total data collection period lasted from February to November 2021. This time period was chosen to ensure sufficient follow-up time of three months after surgery, while not being extended due to restrictions on access to hospitals due to measures for the COVID-19 pandemic.

### 2.5. Intervention Description

The intervention took place three months after the surgery and included communication lasting 10 min. More specifically, the intervention included a set of awareness-raising, psychoeducational, and informational questions and aimed to express emotions, active listening, communication, information, enhance self-efficacy, and mobilize to seek psychosocial support that advocate for better management of the disease.

The intervention was designed based on principles of psychosocial support and included thematic modules aimed at emotional expression, enhancing self-efficacy, and improving family support, such as the following:

A. Expression of emotions

Exploration of how the patient feels about her experience.

B. Active listening and emotional support

Enhancement of trust and empathy.

C. Provision of information

Information on psychological adaptation and available support structures.

D. Enhancement of self-efficacy

Discussion on disease management strategies.

E. Mobilization to seek support

Enhancement of the role of the family and social environment in support.

The objectives of the intervention were as follows:

- Strengthen personal skills.
- Development of adaptation strategies.
- Enhancement of social networking.
- Motivation to seek psychosocial support.

This method was chosen due to its ease of application by health professionals and the possibility of use in remote areas.

The intervention was implemented by the researcher himself to all participants based on the predetermined five-axis protocol, lasting 10 min. To ensure uniformity, a specific manual with a defined series of questions and a common duration was used.

The timing of the intervention (three months after surgery) was chosen based on the gradual recovery of the patients and their entry into a phase of acceptance of the disease and adaptation to the new condition. At this time, the participants should be able to actively process the information of the intervention, express their emotions, and be mobilized to seek psychosocial and family support. This choice enhances the potential effectiveness of the intervention, as it is associated with increased receptivity and the possibility of focused empowerment.

### 2.6. Ethics

This study was conducted in accordance with the ethical standards of the institutional and national research committees, the Declaration of Helsinki (2013), and the EU General Data Protection Regulation (GDPR, 2019).

Approval was granted by the scientific councils of the participating hospitals (Protocol Nos. 555/28-06-2019 and 28666/11-12-2019).

All participants provided written informed consent. Participation was voluntary, and data were anonymized and treated as confidential throughout the study.

Participants were also informed that they retained the right to not participate in the study and to discontinue their participation in it, without any impact on the care provided by their treatment, while the submission of complaints and comments was encouraged.

### 2.7. Statistical Analysis

The SPSS 22.0 statistical program was used and the Pearson correlation test (r) was performed, the paired *t*-test was used to compare the scales between the measurements, the analysis of variance for repeated measures (ANOVA) was used to test differences in the scales before and after the intervention depending on the type of treatment, the degree of change over time of the parameters under study, and the degree of correlation between the changes in the scales were estimated with partial correlations taking into account the treatment.

The internal reliability of the questionnaire was tested using the Cronbach’s alpha coefficient.

## 3. Results

### 3.1. Descriptive Statistics of the Sample

In the present study, the sample consisted of 58 women with breast cancer. The mean age of the women was 52 (SD = 10.2 years) (Table 1).

According to Table 1, the majority of the patients’ educational level was higher education, with 32.8%. The occupational status of the participants with the largest percentage, 29.8%, was public employees, followed by 21.1% for private employees.

Regarding family status, 66.7% of the women were married and the vast majority of the participants were mothers, making up 82.8%.

Finally, regarding the family history of cancer, 55.2% of the patients responded that there was a family history of cancer in their family and, within that, breast cancer made up the largest percentage at a rate of 37.9%.

### 3.2. Changes in the Scales

The following table shows the scores on the scales under study before and after the intervention in these women (Table 2).

It was observed that the scales did not change significantly postoperatively, and there were only minimal changes. A small decrease in self-efficacy and family communication for problem solving was observed and family support improved although there were no statistically significant differences in the scales.

### 3.3. Changes in the Scales According to the Type of Treatment

The changes in the SRCE-GR, FS-12, and FPSC scales according to the type of treatment of the participants are given in the table below (Table 3).

From the above table it can be observed that no statistically significant difference was found in the participants’ scores. The scores did not differ significantly between the types of treatment after the intervention. The degree of change in the scores was similar (minimal differences) across the different types of treatment. More specifically, a small decrease in self-efficacy and family communication for problem solving was observed and family support improved except in the case of radiotherapy.

It should be noted that given the limited sample size, the analyses by treatment type are presented as exploratory results.

### 3.4. Correlations Between the Changes in the Scales After the Intervention

Table 4 below shows the values of the correlations between the scales of the SRCE-GR, FS-12, and FPSC questionnaires.

From the above table it can be observed that the change in the ability to maintain social relationships scale was significantly positively correlated with the change in the ability to solve problems in the family scale (r = 0.59, *p* = 0.043). Therefore, the more the ability to maintain social relationships of the participants improves, the more the ability to solve problems in the family improves.

## 4. Discussion

The present intervention study consists of a sample of 58 breast cancer patients who underwent mastectomy, and their average age was 52 years (SD = 10.2).

This study investigated the effect of a brief digital psychosocial intervention on self-efficacy, family problem-solving communication, and perceived social support in women with breast cancer undergoing mastectomy. The results provide preliminary evidence that even a brief and targeted intervention can enhance critical psychosocial parameters.

Both the changes in the scales before and after the brief intervention and the type of treatment that the patients followed did not significantly affect (small differences) communication for solving family problems, family support, and the ability to maintain social relationships (self-efficacy).

Regarding the patients’ self-efficacy, a small decrease was observed, which was not statistically significant, even though mastectomy and the beginning of treatment intervened from the first to the second measurement.

This finding probably reflects the particular psychosocial conditions of the postoperative period, during which patients are required to adapt physically and emotionally. This decrease may indicate temporary insecurity or uncertainty, despite the intervention. Given that the intervention was of limited duration, it is possible that its content was not sufficient to cause an immediate increase in self-efficacy. This observation highlights the need for longer-term or enhanced interventions.

Self-efficacy has been shown to be critical for the adjustment of breast cancer patients, as it is associated with reduced levels of anxiety, enhanced mental resilience, and improved quality of life [21,22].

A recent study concluded that greater family support for patients increases their self-esteem [29]. Finally, a study indicates a strong relationship between family support and self-efficacy of patients with breast cancer [30]. Regarding family support, the results of the study showed that family support improved little, both during the postoperative period and in relation to the type of treatment that the patients received. The brief intervention was observed to slightly improve patients’ family support, although it was not statistically significant. A recent study suggests that family support was significantly improved in breast cancer survivors [30].

Another finding of this study showed that patients’ self-efficacy after the intervention was significantly associated with patients’ communication to solve family problems. The more a patient’s self-efficacy improves, the more their ability to solve problems in the family improves. This means that patients who were willing to choose problem solving as an adaptive coping mechanism for the disease respond better to the stressors created by the disease and feel more effective. This finding is consistent with similar studies.

More specifically, a study that aimed to examine the relationship between self-efficacy and problem solving (as an adaptive coping mechanism) in cancer patients showed a significant effect of self-efficacy on problem solving. People with high self-efficacy tend to have better problem-solving abilities, which is effective in adapting to the disease [31].

In addition, patients’ self-efficacy is important for solving the problems and consequences created by cancer, and strengthening patients’ low self-efficacy contributes to supporting cancer patients [22].

On a broader level of research, it was found that mastectomy significantly affects (in addition to self-efficacy) both body image and self-esteem, and self-perception of women [32,33,34].

Therefore, self-efficacy, family support, and communication to solve family problems were not significantly affected. It would be expected that both patients’ self-efficacy and family support and communication to solve family problems would have been significantly negatively affected after the disease and the beginning of patients’ treatments. The hypothesis that may support this result is that the brief intervention improved the patients’ ability to maintain social and family relationships after the onset of the disease.

The small variation in results may be attributed to the small number of participants or the short duration of the intervention. However, the significant association between self-efficacy and family communication demonstrates the potential impact of targeted psychosocial interventions.

The study highlights the need for larger interventions that incorporate structured psychosocial strategies. Compared to other studies, self-efficacy after mastectomy is significantly influenced by family support, which is reinforced by the statistically significant SRCE-FPSC correlation.

These findings suggest that even a brief intervention, focused on empathy, psychoeducation, and motivation, can substantially contribute to strengthening patients’ interpersonal resilience.

## 5. Limitations, Strengths and Future Directions of the Study

This study has several limitations, which are, firstly, related to the limitations of the conditions caused by the COVID-19 pandemic. Secondly, the small number of participants does not allow us to conduct further statistical analysis regarding the socio-economic, educational, and cultural level of the participants and may hinder the generalization of the results. Despite the small number of participants, an attempt was made to assess the adequacy of the sample based on previous studies with a similar design. However, an a priori power analysis was not performed. The absence of a priori calculation of sample size may limit the ability to detect small or moderate effects. Although no prior power analysis was performed, the size of 58 participants was deemed sufficient to statistically detect differences within the same sample. The interpretation of the findings is based on both statistical significance and effect sizes. The choice of the sample size was based on practical and ethical criteria, taking into account the limitations of the COVID-19 period.

Thirdly, through the questionnaires, more information could be collected, such as on the quality of life of the patients, cultural elements, etc. Fourthly, the data collection of the sample was performed for one region of Greece.

Finally, a major limitation of this study is the absence of a control group, which limits the possibility of causal interpretation of the changes as a direct result of the intervention. The study was designed to initially assess the applicability of a digital intervention in patients with breast cancer. The choice of a within-group design was deemed more appropriate in the context of the health and operational constraints of the period (COVID-19) and based on ethical criteria. Future studies with randomization and a control group are necessary to confirm the results and strengthen the internal validity.

Furthermore, the sample size does not allow for firm conclusions regarding possible differences by treatment type. The relevant analyses were presented solely as exploratory and require confirmation through larger-scale studies.

In summary, the future directions of the research are as follows:

1. Sample expansion and addition of a control group: studies with a larger number of participants and the use of a control group (RCT) can increase the validity of the results.
2. Assessment of long-term effects: studies that examine the effects at a depth of 6 months or 12 months after the intervention could show whether the effect is stable or transient.
3. Expansion of measurements: addition of tools to assess quality of life, psychological resilience or self-esteem.
4. Adaptation to different populations: the intervention could be adapted to patients with other types of cancer or chronic diseases, to examine the generalizability of the model.
5. Combination of digital and in-person support: possibility of combining the brief digital intervention with guided sessions or support groups.

However, this particular study may be an incentive for new studies in the future. According to the bibliography, a research gap is observed in this scientific field in the most critical period of the disease of breast cancer (preoperative and postoperative period). Finally, it can provide the impetus for the scientific community to develop interventions that can be carried out by all health professionals and through digital means, which are effective, empowering, and promote self-efficacy in breast cancer patients.

Therefore, it is recommended to increase the sample size to improve statistical power, to extend the intervention, and to combine digital and in-person interventions.

## 6. Clinical Implementations of the Study

This study highlighted an important aspect of the vulnerability of the preoperative and postoperative period of breast cancer. During this crucial phase of their illness, patients require more support, and the involvement of health professionals is considered to be especially important.

Through brief interventions, patients can be sensitized, mobilized, and empowered to seek further psychosocial support that alleviates the psychological and physical effects of the disease.

The brief intervention can be integrated as a complementary psychological support service in oncology hospitals, especially during the perioperative period.

The digital intervention of the study can also be used in remote areas where patients do not have access to health facilities or for patients who have difficulty accessing health facilities.

These interventions are quite easy to use by health and mental health professionals and are particularly effective for patients.

Therefore, the digital intervention can be widely implemented, especially in cases where patients have limited access to psychosocial support services, allowing it to be used as a support tool after discharge from the hospital in the context of home care, and with appropriate cultural adaptation, can be used in immigrant women.

In addition, it can be integrated into breast cancer rehabilitation programs after surgery and act preventively for the early management of stress, uncertainty, and fear of recurrence, empowering patients.

Finally, the structure of the intervention could be transformed into a digital application (app) or interactive website, which patients can access without the presence of a professional, enhancing autonomy and reducing costs.

## 7. Conclusions

According to the findings of the study, self-efficacy, communication to solve family problems, and family support were not significantly affected during the postoperative period. The intervention contributed to the mobilization and empowerment of breast cancer patients both in terms of maintaining social and family relationships and in terms of communication to solve intra-family issues. However, improving intra-family communication simultaneously improves patients’ self-efficacy. The present study shows that self-efficacy and family communication are related, but were not significantly changed due to the intervention. The development of more extensive intervention programs could strengthen the results.

## Figures and Tables

**Table 1:** Sociodemographics of the sample.

Variables		Ν	M/Percentage %
Age (SD)		52 (10.2)	
Education level	Primary school	2	3.4
	Middle school	5	8.6
	High school	14	24.1
	Post-secondary education	7	12.1
	Higher education	19	32.8
	Post graduate education	7	12.1
	PhD	4	6.9
Employment Status	Unemployed	5	8.8
	Private employee	12	21.1
	Civil servant	17	29.8
	Housekeeping	9	15.8
	Entrepreneur	1	1.8
	Freelancer	3	5.3
	Other	10	17.5
Area of residence	<2000 inhabitants	4	6.9
	2000–10,000 inhabitants	7	12.1
	10000–100,000 inhabitants	12	20.7
	>100,000 inhabitants	35	60.3
Marital status	Unmarried	6	10.5
	Partnership	1	1.8
	Married	38	66.7
	Divorced	7	12.3
	Separated	2	3.5
	Widowed	3	5.3
Mothers	No	10	17.2
	Yes	48	82.8
Family history for cancer	No	26	44.8
	Yes	32	55.2

**Table 2:** Scores and changes in the scales under study before and after the intervention.

	N	Before	After	Change	Paired *t*-Test
		Μ (SD)	Μ (SD)	Μ (SD)	
Social Relationship Coping Efficacy Scale (SRCE-GR)	58	65.5 (14.2)	63.8 (13.7)	−1.6 (12.5)	0.324
Family support (FS-12)	58	47.5 (7.4)	48.4 (7.3)	0.9 (6.6)	0.338
Family Problem Solving Communication (FPSC)	57	20.3 (4.1)	19.3 (4.4)	−1 (4.2)	0.097

**Table 3:** Changes in the SRCE-GR, FS-12, and FPSC scales according to the type of treatment of the participants.

	Type of Treatment	Before	After	Change		
		M (SD)	M (SD)	M (SD)	*p* ^2^	*p* ^3^
Social Relationship Coping Efficacy Scale (SRCE-GR)	Chemotherapy	66.3 (13.3)	64.1 (15.2)	−2.2 (9.5)	0.267	0.935
	Radiotherapy	71.3 (12)	70.9 (10.2)	−0.4 (14.4)	0.943	
	Hormonetherapy	63.3 (18.9)	58.9 (6.8)	−4.4 (18.9)	0.533	
	None	61.8 (12.9)	60.2 (14.9)	−1.6 (12.8)	0.656	
	***p* ^1^**	0.475	0.266			
Family support (FS-12)	Chemotherapy	49.2 (5.2)	49.6 (5.4)	0.4 (2.8)	0.600	0.473
	Radiotherapy	51.6 (5.2)	51.4 (5.9)	−0.1 (1.9)	0.846	
	Hormonetherapy	45.3 (10.8)	47.9 (10.1)	2.6 (11.5)	0.577	
	None	44.9 (7.5)	48.5 (7.3)	3.6 (6.9)	0.102	
	***p* ^1^**	0.158	0.845			
Family Problem Solving Communication (FPSC)	Chemotherapy	19.1 (4.4)	18.6 (4.2)	−0.5 (3.4)	0.479	0.976
	Radiotherapy	20.9 (3.2)	20.8 (3)	−0.1 (3.8)	0.928	
	Hormonetherapy	21.3 (4.2)	20.9 (5)	−0.4 (3.8)	0.774	
	None	21.2 (2.8)	20.4 (2.8)	−0.8 (2.3)	0.260	
	***p* ^1^**	0.298	0.354			

^1^ Difference between groups; ^2^ difference between measures; ^3^ repeated measures ANOVA. Differences in change between groups from one measure to another.

**Table 4:** Correlations between the changes in the scales SRCE-GR, FS-12, and FPSC after the intervention.

Changes:		Social Relationship Coping Efficacy Scale (SRCE-GR)	Family support (FS-12)	Family Problem Solving Communication (FPSC)
Social Relationship Coping Efficacy Scale (SRCE-GR)	r	1.00	−0.43	0.59
	*p*		0.164	0.043
Family support (FS-12)	r		1.00	−0.41
	*p*			0.181
Family Problem Solving Communication (FPSC)	r			1.00
	*p*			

## Data Availability

Data are not available due to confidentiality agreements with this study’s participants.
